# Post-Ureteroscopy Infections Are Linked to Pre-Operative Stent Dwell Time over Two Months: Outcomes of Three European Endourology Centres

**DOI:** 10.3390/jcm11020310

**Published:** 2022-01-09

**Authors:** Robert M. Geraghty, Amelia Pietropaolo, Luca Villa, John Fitzpatrick, Matthew Shaw, Rajan Veeratterapillay, Alistair Rogers, Eugenio Ventimiglia, Bhaskar K. Somani

**Affiliations:** 1 Department of Urology, Freeman Hospital, Freeman Road, Newcastle-upon-Tyne NE7 7DN, UK; rob.geraghty@newcastle.ac.uk (R.M.G.); john.fitzpatrick4@nhs.net (J.F.); Matthew.shaw7@nhs.net (M.S.); r.veeratterapillay@nhs.net (R.V.); Alistair.rogers2@nhs.net (A.R.); 2 Department of Urology, University Hospital Southampton, Tremona Road, Southampton SO16 6YD, UK; ameliapietr@gmail.com; 3 Division of Experimental Oncology/Unit of Urology, URI, IRCCS Ospedale San Raffaele, 20132 Milan, Italy; villa.luca@hsr.it (L.V.); eugenio.ventimiglia@gmail.com (E.V.)

**Keywords:** ureteroscopy, infection, post-operative, ureteric stent, kidney calculi

## Abstract

Background: The aim of this study is to investigate outcomes of pre-operative stent dwell time on infectious complications following ureteroscopy and stone treatment to identify a time cut-off. Material and Methods: Three tertiary referral centres in Europe retrospectively collected outcomes of ureteroscopy and laser fragmentation (URSL) for all patients with pre-operative indwelling ureteric stents over a period of up to 5 years. Data was collected on patient details, stone demographics, stent dwell time, complications and stone free rate (SFR). Matching for age, sex, operative time, stone size and post-operative stent insertion. To examine for a threshold effect, monthly cut-offs were used to compare post-ureteroscopic febrile UTIs. Binomial logistic regression was used (SPSS v.24) with a significance level set at 0.0036. The risk ratio (RR) with a 95% confidence interval (CI) and the number needed to harm (NNH) are reported. Results: There were 467 patients with a pre-operative stent for analysis. These patients (*n* = 315) were matched to non-stented controls after excluding 152 patients to achieve adequate matching. There was a significant difference in rates of post-ureteroscopic febrile UTI between stented vs non-stented patients (RR = 2.67, 95% CI: 1.10–6.48, *p* = 0.03). On adjustment, a dwell time of more than two months was associated with an increased risk of post-ureteroscopic febrile UTI (RR = 3.94, 95% CI: 1.30–12.01, *p* = 0.02), this increased risk rose with longer dwell time. At stent time longer than four months was associated with a significantly increased risk of post-ureteroscopic febrile UTI (5% vs. 15%, RR = 3.09, 95% CI: 1.56–6.10, *p* = 0.001), with the number needed to harm at 10. Conclusions: Overall infectious complication rates from URSL are low. The risk of post-operative UTI after four months of dwell time is nearly tripled compared to less than four months.

## 1. Introduction

Kidney stone disease (KSD) is becoming an increasingly prevalent problem [1]. Surgical practice has changed over the past two decades and ureteroscopy has become one of the most common interventions for KSD [2]. An important, and therefore investigated post-ureteroscopic complication is urinary tract infection (UTI) [3], which arises in around 5% of all ureteroscopies [4].

Multiple risk factors have been investigated and identified including diabetes mellitus, positive pre-operative urine culture and extended operative time, although no single study demonstrates all of these simultaneously [5]. One of the more consistent risk factors for the development of post-ureteroscopic infection is the presence of a pre-operative stent [4,5,6]. Patients are often stented to drain an obstructed urinary system or to passively dilate a narrow ureter. Although not clearly defined, the interval between stent and definitive ureteroscopy is generally agreed to be as short as possible [5,6,7]. The British Association of Urologists recommend an interval of no more than 4 weeks [8], although this is based on low level evidence [9].

The likelihood of post-ureteroscopic infection in patients with an indwelling stent is 1.5 times more than those without [6]. This quadruples for those with a prior history of urosepsis [4]. With increasing stent dwell time there is an increased likelihood of bacterial colonisation and subsequent bacteriuria [10,11]. Logically, therefore, the longer a stent is in situ the higher the risk of post-ureteroscopic infection. One previous single-centre cross-sectional study demonstrated that patients who developed post-ureteroscopic urosepsis were more likely to have had a longer stent dwell time [12]. However, this study has not described the risk of post-ureteroscopic infection nor a specific time after which infection is more likely.

Our primary aim therefore was to use multi-centre data to examine the risk of post-ureteroscopic infection, defined as febrile urinary tract infection (febrile UTI), for monthly time cut-offs up to 6 months dwell time. With a secondary aim of replicating previous studies examining the risk of febrile UTI in those with against those without an indwelling stent pre-operatively.

## 2. Methods and Materials

### 2.1. Patients

Three European tertiary referral centres contributed retrospectively ascertained data from electronic health records. Each centre collected data on all patients undergoing ureteroscopy (URS) in certain time periods. The first centre was between April 2013 and September 2017, the second between June and September 2019, and the third between December 2015 and November 2020.

Stented patients were matched for age (±1 year) and sex to those without a pre-operative stent.

### 2.2. Variables Collected

Pre-operative variables collected were age, sex, pre-operative urine culture result, stone size (maximum stone diameter), location and pre-stented status/duration. Operative variables: operative time and post-operative stent insertion. Post-operative outcomes: febrile UTI development and urosepsis.

### 2.3. Definitions

“Febrile UTI” was defined as fever >38.0 °C along with symptoms of urinary tract infection (dysuria, haematuria, urinary frequency, urinary urgency and/or malaise).

“Urosepsis” was defined as UTI with two or more points on the SOFA (Sequential Organ Failure Assessment) score as per the Third International Consensus on Sepsis [13,14].

“Maximum stone diameter” was defined as longest measurement in any orientation.

### 2.4. Statistical Analysis

Analysis and graph generation was performed in SPSS (IBM, Armonk, NY, USA, version 24) and R (Vienna, Austria, version 4.02). Continuous variables were assumed to be normally distributed (age, operative time and stone diameter). Binary logistic regression was used for analysis of binary outcomes. The outcome measure was post-ureteroscopic febrile UTI. Sepsis was not investigated due to very low numbers with this outcome. Adjustments was made for age, sex, maximum stone diameter, operative time, pre-operative culture result and stone free status. These are all risk factors for post-ureteroscopic infectious complication [5]. Matching was performed using the “matchit” package in R for age, sex, stone size and post-operative stent insertion. As these are matched, no statistical adjustments are needed for the primary outcome (febrile UTI), the adjustment is incorporated into study design.

Two separate analyses were performed: Firstly, using the matched cohort, of the rates of post-operative UTI in those with and without a pre-operative stent. Secondly, using only pre-stented patients, to identify at which monthly time point risk rose significantly.

Sample size was calculated at *n* = 75 in each group to show a 15% difference in rate of febrile UTI (5% vs. 20%), with alpha = 0.05 and beta = 0.8. Output was presented as risk ratios (RR) with 95% confidence intervals (CI) and *p*-values. Bonferroni correction was applied to all outcomes to account for multiple significance tests (*n* = 14 tests, therefore significance level set at *p* = 0.0036). Outcome proportions were used to calculate number needed to harm (NNH).

## 3. Results

### 3.1. Patient/Stone Demographics

There were 467 patients undergoing URS with a pre-operative stent in the study period. Stented patients were age and sex-matched to those without a pre-operative stent from the same database. To achieve adequate pairing, 152 stented patients were excluded from the stent vs no stent analysis. Demographics of unmatched stented (*n* = 467) and matched stented/non-stented patients (*n* = 315, in each group) are detailed in Table 1. There was no significant difference in UTI rates between centres (chi-square *p* = 0.67).

### 3.2. Analysis of Non-Stented vs. Stented Patients

There was a significant difference between rates of febrile UTI between not stented (2%) and stented patients (6%) on binary logistic regression (RR = 2.67, 95% CI: 1.10–6.48, *p* = 0.03).

### 3.3. Analysis of Stent Dwelling Time

**Figure 1:**
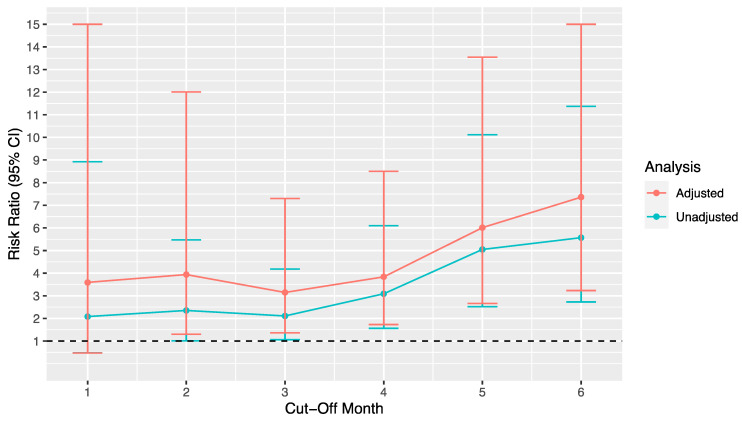
Plot of risk ratios for adjusted and unadjusted risk of post-ureteroscopic febrile UTI by stent dwell time cut-off month (e.g., <1 vs. >1 month). Error bars = 95% confidence interval. Reference line = 1, i.e., no difference in risk. = *p* < 0.0036 as per Bonferroni correction.

**Figure 2:**
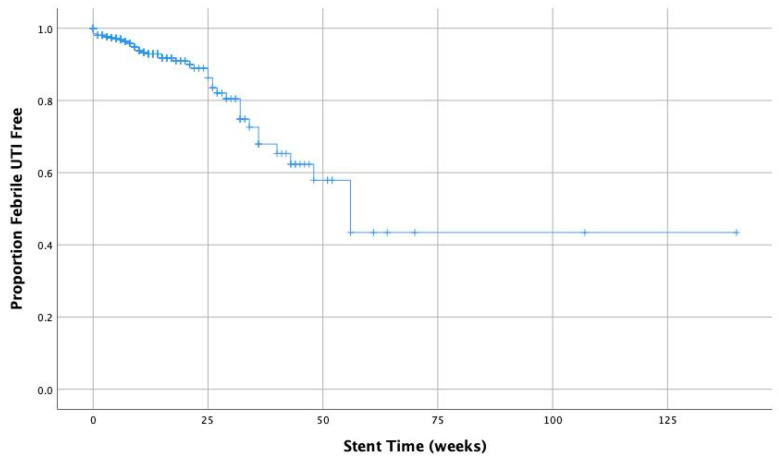
Kaplan–Meier curve for stent dwell time and proportion post-operative febrile UTI free.

The following analyses were conducted using the unmatched stented patients’ data only. In the unadjusted analysis, a stent time longer than 4 months was associated with a significantly increased risk in post-ureteroscopic febrile UTI (5% vs. 15%, RR = 3.09, 95% CI: 1.56–6.10, *p* = 0.001). This risk rose with increasing stent dwell time (see Table 2 and Figure 1 and Figure 2). The number needed to harm at 4 months dwell time was 10.

On adjustment, dwell time of more than 2 months was associated with an increased risk of post-ureteroscopic febrile UTI (RR = 3.94, 95% CI: 1.30–12.01, *p* = 0.02); this increased risk rose with longer dwell time. The number needed to harm fell with increasing stent dwell time, reflecting the increase in risk.

## 4. Discussion

This study replicated previous analyses indicating that the presence of a pre-operative stent is a risk factor for post-operative febrile UTI. Further to this, we demonstrated that longer stent dwell time proportionally increases the risk of post-ureteroscopic febrile urinary tract infection (UTI). A stent dwell time of more than 4 months is associated with a significantly increased risk of UTI in both unadjusted and adjusted analyses, but in adjusted analyses only, the first is significant at 2 months. This risk rises as the number needed to harm falls with increasing time. 

The main strength of this study lies in the large number of stented patients available for analysis, with appropriately powered and robust analyses corrected for multiple testing. We clearly identify that after 4 months stent dwell time, the risk of post-ureteroscopic febrile UTI rises significantly. The overall rates of febrile UTI and sepsis are comparable to previous studies; this study is therefore representative [12,15].

The main limitations of this study are its retrospective nature and lack of other significant risk factors that contribute to febrile UTI. We have included as many potential risk factors as were available, but others such as having diabetes mellitus [15], a neurogenic bladder [16] or data on why the patient had been stented pre-operatively [17] were not complete, considering those stented for infection have a higher risk of infectious complication. However, the risk factors we have included are consistently reported in the literature [5], whilst those factors we have not included have been reported in single studies only. There is clear heterogeneity in the literature on this subject.

There has been only one previous study on stent dwell time [12]. Nevo et al. demonstrate the increased likelihood of post-ureteroscopic sepsis with increasing stent dwelling time. They examined a cut-off of 30 days (albeit not statistically), demonstrating a five-fold increase in rates of sepsis after this time point. The significance of this finding is not clear, as 75% of their patients had stent dwell times of 37 days or longer, implying that few patients had shorter dwell times. It is therefore unclear exactly how long the stent dwell time was for those that developed sepsis. As the authors concede, in a public health system with limited resources it is not always possible to have prompt definitive surgery. Therefore, defining a distinct cut-off, as we have, is key for resource prioritisation. This leads into a wider point concerning stent management within public health systems. In the NHS the approach to stent management is heterogenous, as highlighted by the recent “Getting It Right First Time” (GIRFT) report [18]. A subsequent, related report by the Healthcare Safety Investigation Branch, also highlights the need for a more robust, homogenous approach to stent management. Failure to do so results in significant morbidity suffered by patients [19].

Our observation that the risk of post-ureteroscopic febrile UTI rises after 4 months correlates with a recent study demonstrating that stent encrustation rates rise markedly after 4 months in stone formers [20]. In this study, we did not look at other complications of indwelling stent or surgery itself, and this could influence the risk of infectious complications also. Encrustation is not only a risk factor for bacteriuria [21], but also increases operative time [22], another risk factor for post-ureteroscopic infection [5]. Future studies should examine this effect prospectively. Modern machine learning techniques could be applied to a larger dataset to build predictive models to identify patients who could develop a post-ureteroscopic febrile UTI.

## 5. Conclusions

There is a significantly increased risk of post-operative febrile UTI after two months stent dwell time (6% vs. 14%). This risk is robust to adjustment, increases as dwell time increases, and is independent of other operative factors. We recommend that timely treatment for stented patients within four months should be prioritised.

## Figures and Tables

**Table 1:** Patient demographics in the study.

		Matched No Pre-Operative Stent (*n* = 315)	Matched Pre-Operative Stent (*n* = 315)	Total with Pre-Operative Stent (*n* = 467)
**Mean Age ± SD**		57 ± 17	57 ± 18	58 ± 16
**Male, *n* (%)**		172 (54.6%)	172 (54.6%)	285 (61%)
**Mean Operative Time (mins) ± SD**		45 ± 26	48 ± 26	51 ± 28
**Mean Stone size, (mm) ± SD**		10 ± 5	10 ± 5	10 ± 6
**Patients with Multiple stones, *n* (%)**		140 (44%)	113 (36%)	181 (37%)
**Post-operative stent insertion, *n* (%)**		264 (84%)	267 (85%)	428 (86%)
**Stone Location, *n* (%)**	**Renal**	225 (71%)	169 (54%)	192 (38%)
	**Proximal ureter**	30 (10%)	71 (23%)	74 (15%)
	**Mid ureter**	18 (6%)	35 (11%)	59 (12%)
	**Distal ureter**	29 (9%)	34 (11%)	117 (23%)
	**VUJ**	13 (4%)	6 (2%)	57 (11%)
**Positive Pre-operative Urine culture, *n* (%)**		31 (10%)	81 (26%)	175 (38%)
**Post-operative Febrile UTI, *n* (%)**		7 (2%)	18 (6%)	37 (8%)
**Post-operative Urosepsis, *n* (%)**		3 (1%)	5 (2%)	6 (1.3%)

**Table 2:** Risk of post-ureteroscopic febrile UTI. Adjustments for age, sex, maximum stone diameter, operative time, positive pre-operative urine culture and stone free status. RR = Risk ratio, CI = confidence interval. Significance level = 0.0306.

Cut-Off Month	Febrile UTI, *n* (%)		Number Needed to Harm	Unadjusted		Adjusted	
	<Specified Month Cut-Off	>Specified Month Cut-Off		RR (95% CI)	*p*	RR (95% CI)	*p*
1	2 (4.3%)	35 (8.5%)	24	2.08 (0.48–8.93)	0.33	3.59 (0.47–27.72)	0.22
2	7 (4.5%)	30 (9.9%)	19	2.35 (1.01–5.47)	0.05	3.94 (1.30–12.01)	0.02
3	15 (5.7%)	22 (11.2%)	18	2.11 (1.06–4.18)	0.03	3.15 (1.36–7.30)	0.007
4	18 (5.4%)	19 (15.0%)	10	3.09 (1.56–6.10)	0.001	3.84 (1.73–8.50)	<0.001
5	19 (5.1%)	18 (21.2%)	6	5.05 (2.52–10.12)	<0.001	6.01 (2.66–13.55)	<0.001
6	21 (5.3%)	16 (23.9%)	5	5.57 (2.73–11.37)	<0.001	7.36 (3.23–16.78)	<0.001

## Data Availability

As data is identifiable it will not be made available as per ethical approval.
